# Mate-pair editing: a perspective to double mate-pair sequencing coverage

**DOI:** 10.1007/s13205-016-0554-z

**Published:** 2016-10-31

**Authors:** Abdulqader Jighly

**Affiliations:** School of Applied Systems Biology, La Trobe University, Bundoora, VIC 3083 Australia

**Keywords:** Next-generation sequencing, Mate-pair library

## Abstract

In this report, I am proposing a hypothetical approach that can enable sequencing of four short reads from the same insert using Illumina next-generation sequencing. The methodology is similar to that used in mate-pair sequencing except that it involves two circularization steps and the sequencing slide should have four different oligonucleotides.

## Background

Mate-pair (MP) libraries enable two distanced (2–5 kb or more) small DNA fragments to be sequences as a pair of short reads in next-generation sequencing systems. This can simplify complex applications like ordering contigs in de novo assembly, detecting structural variants and targeting large rearrangements. MP library preparation involves a circularization of large DNA sequences to position the edge distal fragments adjacently. Thus, they are distinguished from other libraries with their large insert size between the sequenced fragments (Metzker 2010).

In this perspective report, I am proposing a new design for sequencing library similar to MP library named mate-pair editing. Combined with some modifications to the standard Illumina sequencing procedure, this methodology can gain more coverage within the MP insert by the ability to sequence four fragments instead of the two fragments targeted by the original MP procedure. The main idea is to have an extra circularization step where an additional unique sequence in the ligation site can be added. This extra sequence should be recognized by an engineered nuclease which can only cut the DNA using one of the genome editing technologies (Kim and Kim 2014).

**Fig. 1:**
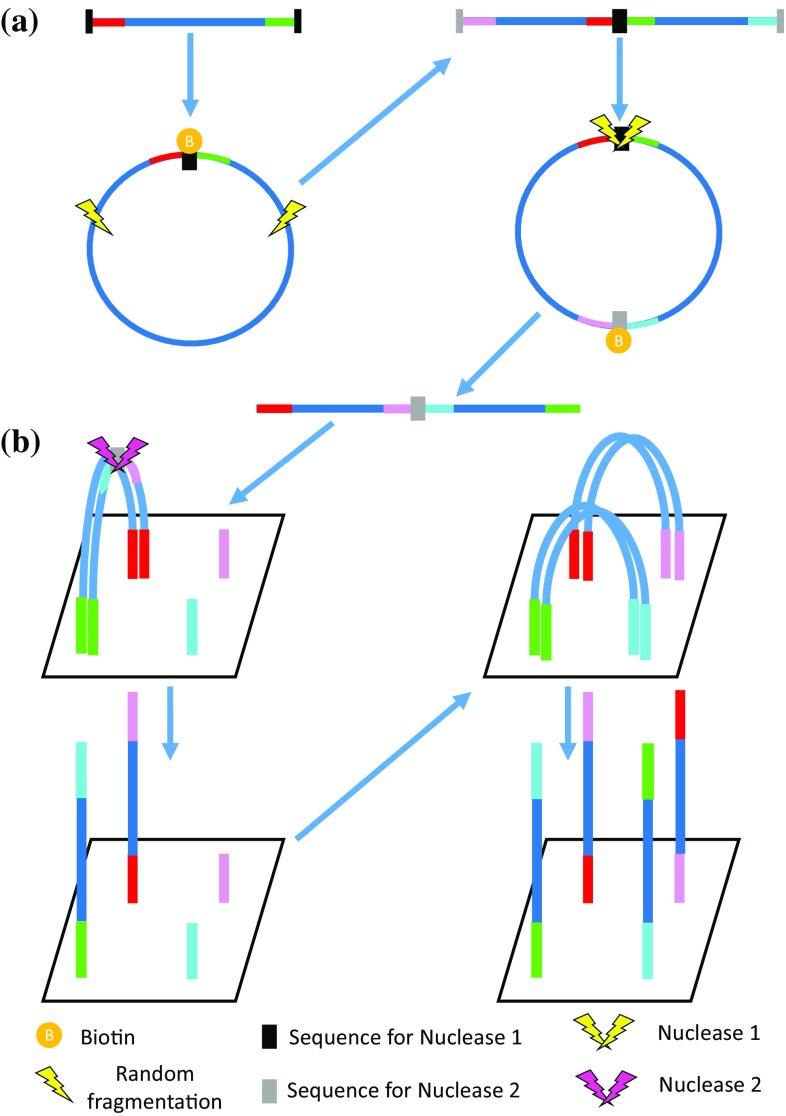
Steps of the suggested methodology.** a** Library preparation** b** design for the sequencing slide

Figure 1a illustrates the hypothetical procedure to generate the MP editing libraries. For MP editing, the adapters should have an extra short oligonucleotide that can be distinguished by nucleases. Starting with the same steps as the standard MP protocol until ligating adapters after circularization, we can then start a second round of circularization with another set of unique adapters that have nuclease 2 target sequence. This time the fragmentation should be directed toward the first circularization ligation point since we added a nuclease 1 target sequence. The library preparation stage will end with a sequence flanked by two adapters complementary to the amplification oligonucleotides on the sequencing slide. Moreover, there is a nuclease 2 target sequence in the middle of the fragment flanked by two unique adapters. The amplification procedure on the sequencing instrument for MP editing is illustrated in Fig. 1b. The slide should be adjusted to complement the four used adapters. After the first bridge amplification and before the denaturation, nuclease 2 can be used leaving two fragments each one flanked by two different adapters. The following bridge amplification will end with two different strands and their complementary sequences which should grow in the following cycles to form the sequencing cluster.

In standard Illumina sequencing, only one strand can be kept on the slide while the complementary strand should be cleaved and washed away during the sequencing stage to ensure that all copies are sequenced in the same direction from the same end (http://nextgen.mgh.harvard.edu/IlluminaChemistry.html). The reverse strands for both fragments should be washed away, then the free 3′ ends should be blocked to prevent unwanted DNA priming. Thus, the sequencing primers should not have any complementary sequences between fragment one and two as both fragments forward sequences will be found on the slide at the same time. The first round of sequencing should cover the first fragment forward direction. A washing step should follow sequencing read 1 in order to start with read 2 sequencing, the forward direction for the second fragment. After completing read 2 sequencing, a bridge amplification followed by cleaving the forward strands should be done in order to sequence read 3 (reverse direction for the first fragment) and read 4 (reverse direction for the second fragment).

## Conclusion

Theoretically, the methodology described here should be applicable. If so, it can be further improved and optimized to have three or more circularization steps in order to sequence more than four fragments. Although Illumina has recently developed the synthetic long reads which can limit the use of MP libraries, the cost per Gb for this technology is about 1000$ (Goodwin et al. 2016) which is much higher than the standard Illumina short-read protocols.

## Abbreviation

MP: Mate-pair
